# Cycling in people with a lower limb amputation

**DOI:** 10.1186/s13102-021-00302-3

**Published:** 2021-07-10

**Authors:** Jutamanee Poonsiri, Rienk Dekker, Pieter U. Dijkstra, Juha M. Hijmans, Jan H. B. Geertzen

**Affiliations:** 1 Department of Rehabilitation Medicine, University of Groningen, University Medical Center Groningen, Groningen, The Netherlands; 2 Sirindhorn School of Prosthetics and Orthotics, Faculty of Medicine Siriraj Hospital, Mahidol University, Bangkok, Thailand; 3 Department of Oral and Maxillofacial Surgery, University of Groningen, University Medical Center Groningen, Groningen, The Netherlands

**Keywords:** Cycling, Sport, Transport, Recreation, Amputees, Prostheses, Barriers, Facilitators

## Abstract

**Background:**

To evaluate cycling participation and identify barriers and facilitators related to cycling participation in people with a lower limb amputation (LLA) in the Netherlands.

**Methods:**

A questionnaire was sent to adults with a LLA between March and August 2019 to obtain information regarding prosthesis, individual’s characteristics, amputation, cycling barriers and facilitators, and prosthetic satisfaction. The questionnaires were distributed via 8 orthopedic workshops, post and were given directly. To find cycling predictors, variables associated with cycling (*p* < 0.1) were entered into a logistic regression analysis. Non-significant variables were removed manually.

**Results:**

Participants (n = 207, 71% males) had a mean age of 62.0 ± 13.0 years. The most frequent level of amputation was transtibial (42%), and trauma was the most frequent cause of amputation (43%). After the LLA, 141 participants (68%) cycled for recreation (80%), physical fitness (74%), and transport (50%). In the past six months, cyclists cycled for recreation (79%) and transport (66%). Most cycled less than once a day. Recreational cyclists cycled alone (75%) for a median duration of 45 min or 14 km per ride. Cyclists with a transportation purpose usually cycled to go shopping (80%) or to visit friends (68%), with a median duration of 20 min or five kilometers per ride. Cyclists reported more facilitators (median (IQR) = 5 (3, 7) than non-cyclists 0 (0, 3). The majority of cyclists reported a positive attitude toward cycling (89%) and cycled because of health benefits (81%). A dynamic foot (odds ratio: 5.2, 95% CI 2.0, 13.3) and a higher number of facilitators (odds ratio: 1.3, 95% CI 1.2, 1.5) positively predicted cycling, whereas the presence of other underlying diseases (odds ratio: 0.4, 95% CI 0.2, 0.9) negatively predicted cycling (R^2^: 40.2%).

**Conclusion:**

In the Netherlands, the majority of adults cycled after a LLA, mainly for recreational purposes. A dynamic foot, a higher number of facilitators, and no other underlying diseases increases the likelihood of cycling after a LLA. The results suggest that personal motivation and a higher mobility level could be the key to increasing cycling participation. Future research should determine the association between motivation, mobility levels, and cycling with a LLA.

**Supplementary Information:**

The online version contains supplementary material available at 10.1186/s13102-021-00302-3.

## Background

In many countries, cycling has been integrated into daily life for the purposes of sport, exercise, and transportation. The health benefits of cycling have been studied in able-bodied individuals [1, 2], and a lower incidence of type 2 diabetes [2], coronary heart disease, and mortality from cardiovascular diseases and cancer has been observed in cyclists [1]. Given its numerous health benefits, cycling can be used for the purpose of health promotion [3]. After a lower limb amputation (LLA), people generally become more sedentary [4]. However, cycling remains an activity they can and do partake in [4–6]. Consequently, cycling may also promote the health and wellbeing of people with a LLA. To date, however, only few studies have explored cycling participation in this population [7].

Factors that influence cycling in people with a LLA have been studied mostly in competitive cyclists and were limited to technical factors [7]. These studies showed how prosthetic or bicycle components affect cycling symmetry between the amputated limb and intact limb [8, 9]. Although decreasing the asymmetry between the intact and amputated limb benefits competitive cyclists [8, 9], the results may not be applicable to the majority of recreational cyclists [5, 7]. A study in recreational cyclists with a LLA in Thailand found that a perceived poor health condition was the most important barrier to cycling after a LLA [5]. In contrast, having cycling experience before a LLA was the most important predictor of cycling after a LLA [5]. Thailand environment is different from the Netherlands such as road structure, traffic lights, or weather. Moreover, Thai people may not transport by bicycle as much as Dutch people. Therefore, facilitating factors for cycling in Dutch people with a LLA might differ from those of Thai people with a LLA.

Cycling accounts for more than 25% of all trips in the Netherlands, making it the number one cycling nation [10]. Dutch people with a LLA reported that cycling helped them achieve an active lifestyle [4–6]. It is not yet known how many Dutch people with a LLA cycle and what factors influence cycling participation. Therefore, this study aimed to survey cycling participation (rate, frequency, duration, and reason) and cycling predictors in people with a LLA in the Netherlands. A secondary aim was to identify cycling barriers and facilitators and to identify types of prosthetic devices, bicycles, and shoes used by people with LLA during cycling.

## Methods

### Participants

This cross-sectional survey was conducted from March to August 2019. The sample size was calculated by using a formula suggested for survey studies [11] and this formula was also used in a prior study performed in Thailand [5]. The estimated percentage of cyclists was 50%, with a margin of error of 5% [11] and 10% missing data. As a result, a sample size of 424 participants was aimed at. Participants were included if they: (1) were aged 18 years or older; (2) had undergone a unilateral or bilateral LLA at least 6 months ago; (3) had a LLA ranging from midfoot to hemipelvectomy level; and (4) were able to read and write in the Dutch language. Eligible participants’ addresses were obtained from a previous research database of another study in which people with a LLA had provided consent for future research (n = 259). This database was limited to participants with a LLA who were recruited via Orthopedic workshops (OIM). A sealed envelope was sent to eligible participants, containing the questionnaire and a stamped return envelope. Twenty-five questionnaires were given to eligible participants during the annual meeting of people with a LLA in Amersfoort. In addition, 240 questionnaires were given to eight orthopedic workshops that agreed to participate in the study (30 per workshop). These workshops were located all over the Netherlands including Amsterdam, Arnhem, Breda, Haren, Hoogeveen, Leeuwarden, Ubbergen, Zwolle. The Medical Ethics Assessment Committee for Research of the University Medical Center of Groningen (protocol number = 201800094, METc 2018/079) granted a waiver of consent prior to data collection. The STROBE guidelines were used to report the study results [12] (Additional file 1: STROBE Statement—Checklist of items that should be included in reports of cross-sectional studies).

### Measures

The questionnaire was written in English by the first author for the survey conducted in Thailand [5] (Additional file 2: Questionnaire). The questionnaire consists of six parts. Part 1, 22 items, was developed and used previously to obtain information concerning daily prosthesis, shoes and walking aids. For part 2, general information (8 items), and part 3, amputation information (3 items), questions were taken and modified from the questionnaires of sport participation in people with lower limb amputation and visual impairments [13]. Part 4, cycling participation information (5 items), consisted of information such as cycling frequency, reasons, intensity. Part 5, questions regarding cycling barriers and facilitators, were adapted from the validated and reliable questionnaires of Barriers to Physical Activity Questionnaire for people with Mobility Impairments (BPAQ_MI) and Barriers to Physical Activity and Disability Survey (B-PADs). B-PADs was chosen because cycling is a type of physical activity. In addition, cycling barriers and facilitators from other cycling studies that were not mentioned in the BPAQ_MI [14] and B-PADs [15] were added. Finally, Orthotics and Prosthetics User Survey (OPUS) which is already available and validated [16] for determining satisfaction is added to part 6 of the questionnaire. Before the questionnaire was used, its content validity was assessed by four physicians in rehabilitation medicine. Items with a content validity less than 0.7 [17] were revised.

Next, it was translated into Dutch, after which the questionnaire was translated back into English by means of back translation [18]. Due to discrepancies between the back translation and the original English version, the Dutch version was adjusted until the discrepancies were resolved. For example, “What is/are the barrier(s) for you to ride a bicycle?” was firstly translated to “Wat zijn de beperkingen voor het fietsen voor u? (What are the cycling restrictions for you?)” and finally was adjusted to be “Wat zijn de hindernissen/barrières voor het fietsen voor u? (What are the obstacles/barriers to cycle for you?)”.

In our prior study [5], the prosthetists filled out the information about prosthetic components for each participant at the clinic and provided assistance when the participants had questions about the questionnaires. In this study, participants filled out the questionnaires themselves at home. An experienced Dutch prosthetist adjusted the questions related to prosthetic components, component pictures, and wording to ensure that the participants were familiar with the components and terminology. To verify that participants were capable of answering questions themselves, six people with a LLA pilot tested the questionnaire.

After reviewing feedback from the first pilot test, wording was adjusted before six others completed a second pilot test. Once the feedback from the second pilot test was satisfactory, no further alterations were made to the questionnaire.

### Data analysis

Participants’ characteristics and participation levels were analyzed using descriptive statistics. Categorical variables were expressed as numbers, and percentages and continuous variables were expressed as mean and standard deviation (SD) or median and interquartile range (IQR), depending on the distribution of the data. Items on barriers and facilitators were grouped into different domains based on the BPAQ_MI [19], and a previous study [5]. When a participant selected more than one item from the same subgroup of facilitators or barriers, these items were analyzed as one barrier of facilitator. For example, when a participant reported on three health-related items (e.g., lack of energy, pain, and wound) these three items were counted as one barrier in the health domain.

All factors were analyzed in order to explore their association with cycling. People who cycled after a LLA were classified as cyclists and who did not, non-cyclists. The differences among cyclists and non-cyclists in each factor were analyzed by using independent samples t-test for normally distributed interval data or Mann–Whitney U test for not normally distributed interval data. Chi-square test was used for categorical data. OPUS total score of satisfaction with prosthesis and service were compared between cyclists and non-cyclists by independent samples t-test. Factors associated with cycling at univariate level analysis (*p* < 0.10) were entered into the logistic regression analysis. This p-level at univariate analysis was 0.1 because using 0.05 can fail to identify variables known to be important. The variables that did not increase the model fit significantly or that had a non-significant regression coefficient (*p* > 0.05) were manually removed from the model.

## Results

### Participants

A total of 207 persons (mean age 62.0 ± 13.0, 71% males) returned the questionnaire, of whom 141 cycled after their LLA (68.1%) (95% CI 61.5–74.1). Half of the participants had undergone their LLA at least 13 years ago. Forty-two percent were retired, and 35% were employed. A majority of participants had at least a high school or vocational level education (61%), with a monthly income of 1500–3000 euros (33%), and 53% lived with their partner. The main reason for LLA was trauma (43%). Half of the participants had undergone a transtibial amputation (50%) and did not use walking aids (50%). Only a few participants (3%) had undergone a bilateral LLA, and 3% had undergone osseointegration (Table 1).

**Table 1:** Participant characteristics (n = 207)

Characteristic (number of valid observations)	Mean (SD)	Range (min–max)
Age (years) (n = 207)	62.0 (13.0)	(25.0–94.0)
Body weight (kg) (n = 207)	86.1 (19.4)	(38.0–180.0)
Height (cm) (n = 207)	177.6 (9.6)	(150.0–200.0)
Body mass index (kg/m^2^) (n = 207)	26.9 (5.0)	(14.1–52.0)
Years since amputation (years) (median (IQR)) (n = 207)	13.0 (3.1, 33.2)	(0–72.0)

	n	%
Sex (n = 205)		
Female	59	29
Male	146	71
Living circumstances (n = 207)		
Single	46	22
With parents	2	1
Couple with children	43	21
Couple without children	110	53
Group	1	1
Other	5	2
Highest education level (n = 206)		
No/not finished high school	11	5
High school/vocational school	125	61
Higher education/university	58	28
Other	12	6
Employment status (n = 207)		
Employed	73	35
Unemployed	44	21
Retired	87	42
Other	3	1
Monthly income (Euro) (n = 207)		
Do not wish to answer	54	26
Under 1500 Euros	29	14
1500–3000 Euros	68	33
3001–4500 Euros	44	21
More than 4500 Euros	12	6
Reason of amputation* (n = 200)		
Trauma	85	43
Vascular	35	18
Cancer	27	14
Diabetes	13	7
Congenital	4	2
Other	36	18
Presence of underlying disease besides the cause of amputation (n = 200)^#^	112	56
Rheumatoid arthritis	7	4
Cardiovascular disease	40	20
Diabetes	17	9
Osteoarthritis	31	16
Osteoporosis	6	3
Bronchitis	16	8
Kidney diseases	4	2
Bilateral LLA	6	3
Osseointegration	7	3
Level of unilateral LLA (n = 201)		
Hemipelvectomy	1	1
Hip disarticulation	3	2
Transfemoral	70	35
Knee disarticulation	19	10
Transtibial	101	50
Ankle disarticulation	3	2
Midfoot	1	1
Van Nes rotation plasty	3	2
Cycled before amputation (n = 205)	187	91
Cycles after amputation (n = 207)	141	68
Use of a walking aid other than a prosthesis (n = 207)	103	50
Types of walking aids (n = 103)		
Cane	30	15
Elbow crutch	41	20
Rollator	28	14
Other	4	2

Levels of six persons with a bilateral amputation are: 1 bilateral transfemoral, 2 bilateral transtibial, right transtibial with left midfoot, 1 bilateral ankle disarticulation, and 1 bilateral midfoot

IQR = interquartile range; kg = kilogram; cm = centimeter; m = meter. Valid observations = number of participants answering the question, SD = Standard Deviation

*Reasons of amputation in unilateral cases

^#^Only diseases frequently report (> 1) were mentioned in the table.

### Cyclists’ characteristics and factors associated with cycling

Compared with cyclists, non-cyclists were older and more often retired; more often lived alone; more often had a lower income; more often had underlying diseases; and more often had undergone an amputation for vascular reasons. Of the cyclists, 96% had cycled before the LLA. Of the non-cyclists, this was 82% (p = 0.002). Cyclists less often used a walking aid compared with non-cyclists. Cyclists mostly used a dynamic foot (64%), followed by a SACH (solid ankle cushioned heel) foot (21%). Cyclists reported a smaller total number of barriers and a larger total number of facilitators than non-cyclists (p < 0.001) (Table 2).

**Table 2:** Characteristics of cyclists and non-cyclists

Characteristic (cyclists/non-cyclists)	Cyclist (n = 141)		Non-cyclists (n = 66)		Test statistic	*p*	95% CI
	Mean	SD	Mean	SD			
Age (years) (140/65)	58.8	12.8	69.1	10.3	− 5.71	< 0.001	− 10.4 (− 13.9, − 6.8)
Body weight (kg) (140/65)	85.5	19.7	87.5	19.0	− 0.66	0.508	− 1.9 (− 7.7, 3.8)
Height (cm) (136/59)	178.2	9.6	176.0	9.3	1.47	0.143	2.2 (− 0.7, 5.1)
Body mass index (kg/m^2^) (136/59)	26.5	5.0	28.0	5.0	− 1.89	0.060	− 1.5 (− 3.0, 0.1)
Years since amputation (138/53)	21.9	19.4	14.4	17.0	2.60	0.011	7.4 (1.8, 13.1)
Prosthetic weight (kg) (87/39)	4.6	7.4	3.6	1.7	− 0.87	0.385	1.0 (− 1.3, 3.4)
Satisfaction with prosthesis (133/56)	3.8	0.6	3.8	0.7	0.21	0.835	0 (− 0.2, 0.2)
Satisfaction with prosthetic service (125/50)	4.2	0.6	4.2	0.6	0.04	0.966	0 (− 0.2, 0.2)

	n	%	n	%	Test statistic	*p*
Sex (140/65)						
Female	41	29	18	28	0.06	0.869
Male	99	71	47	72		
Living circumstances (141/66)						
Single	22	16	24	36	11.21	0.001
Other	119	84	42	64		
Highest education level (134/60)						
≤ High school/vocational	89	66	47	78	2.81	0.126
Higher education/university	45	34	13	22		
Employment status (139/65)						
Employed	64	46	9	14	25.30	< 0.001
Unemployed	31	22	13	20		
Retired	44	32	43	66		
Monthly income (Euro) (102/51)						
Under 1500 Euros	15	15	14	28	10.54	0.014
1500–3000 Euros	41	40	27	53		
3001–4500 Euros	37	36	7	14		
More than 4500 Euros	9	9	3	6		
Presence of underlying disease besides the cause of amputation (138/62):						
Yes	67	49	45	73	10.03	0.002
No	71	51	17	27		
Rheumatoid arthritis	2	1	5	4	0.002	0.035
Cardiovascular disease	23	17	17	12	0.109	0.131
Diabetes	6	4	11	8	0.002	0.004
Osteoarthritis	21	15	10	7	0.961	1.000
Osteoporosis	3	2	3	2	0.334	0.386
Bronchitis	8	6	8	6	0.106	0.160
Kidney diseases	2	1	2	1	0.432	0.594
Level of unilateral amputation* (136/62)						
KD, TF, HD	59	43	34	55	2.23	0.167
TT, AD, MF	77	57	28	45		
Uni/bilat (141/66)						
Unilateral	139	99	62	94	3.44	0.064
Bilateral*	2	1	4	6		
Reason of amputation** (119/54)						
Vascular/diabetes	25	21	31	57	21.91	< 0.001
Trauma	67	56	18	33		
Cancer	23	19	4	7		
Congenital	4	3	1	2		
Cycled before amputation (139/66)						
Yes	133	96	54	82	10.74	0.002
No	6	4	12	18		
Use of a walking aid other than a prosthesis (141/66)						
Yes	60	43	43	65	9.18	0.002
No	81	57	23	35		
Use of a walking aid other than a prosthesis (60/43)						
Cane	18	30	12	28	20.46	< 0.001
Elbow Crutch	33	55	8	19		
Rollator	8	13	20	47		
Other	1	2	3	7		
Prosthetic foot (124/50)						
SACH	26	21	23	47	21.91	< 0.001
Single axis	18	15	14	29		
Dynamic foot	78	64	12	25		
TT, AD, MF socket (71/25)						
Thigh corset	4	6	2	8	0.56	0.811
PTBSC/PTBSCSP	38	54	12	48		
PTB	29	41	11	44		
TT, AD, MF liner (68/19)						
Foam	12	18	3	16	1.46	0.557
Polyurethane/silicone	51	75	13	68		
More than 1 type of liner	5	7	3	16		
TT, AD, MF suspension (73/23)						
Seal in liner/silicone ring	7	10	1	4	6.81	0.420
Vacuum	1	1	2	9		
Pin	23	32	5	22		
Straps	4	6	2	9		
Sleeve/elastic	27	37	8	35		
Self-suspension socket	5	7	4	17		
Sleeve and liner ring	5	7	1	4		
Sleeve and pin	1	1	0	0		
HD, TF, KD socket (40/27)						
Flexible Ischial containment	2	5	3	11	2.93	0.760
Flexible Quadrilateral	1	2	1	4		
Hard Ischial containment	19	44	15	54		
Hard Quadrilateral	6	14	4	14		
Flexible KD	9	21	3	11		
Hard KD	3	7	1	4		
HD, TF, KD liner (44/26)						
None	12	27	3	12	2.82	0.102
Foam	3	7	1	4		
Polyurethane/silicone liner	29	66	22	85		
HD, TF, KD knee (46/24)						
Manual knee lock	7	15	6	25	4.33	0.227
Mechanical polycentric	13	28	10	42		
Pneumatic/hydraulic	8	17	4	17		
Microprocessor	18	39	4	17		
HD, TF, KD suspension (77/28)						
Seal in liner/silicone ring	10	21	11	39	5.18	0.060
Vacuum	16	34	9	32		
Pin	5	11	4	14		
Straps	1	2	0	0		
Sleeve	2	4	0	0		
Self-suspension socket	12	26	4	14		
Sleeve and liner ring	1	2	0	0		

	Median (IQR)	Mean rank	Median (IQR)	Mean rank	Test statistic	*p*
Number of barriers (141/66)	0 (0, 2)	93.9	1 (0, 3)	125.6	3226.0	< 0.001
Number of facilitators (141/66)	5 (3, 7)	126.5	0 (0, 3)	55.9	7825.5	< 0.001

CI = confidence interval; kg = kilogram; cm = centimeter; m = meter; IQR = Interquartile Range; HD = hip disarticulation, TF = transfemoral, KD = knee disarticulation, TT = transtibial, AD = ankle disarticulation, MF = midfoot, SACH = Solid ankle cushioned heel, PTB = patellar tendon bearing; PTB-SC = PTB and supra condylar; PTB-SCSP = PTB-SC and suprapatellar

*Two cyclists had undergone a bilateral midfoot amputation and a bilateral ankle disarticulation, respectively. Four non-cyclists, one had undergone a bilateral transfemoral, two had a bilateral transtibial and one had a transtibial and midfoot amputation, respectively

**Reasons for amputation in people with a bilateral amputation were counted as one if reasons left and right were the same. One person with a bilateral amputation had undergone amputation due to trauma and diabetes for the right and left sides, and the reason for amputation was analyzed as diabetes for the univariate analysis

### Reasons for cycling

Most participants who cycled (88%) reported themselves as the person who motivated them to cycle after a LLA. Some participants cycled because their physiotherapist (24%), family (23%), doctor (17%), prosthetist (9%), occupational therapist (6), or friend(s) (6%) had told them to do so (Additional file 3: Motivators and reasons for cycling). Popular reasons for cycling were recreation (80%), increasing or maintaining physical fitness (74%), and transport (50%) (Additional file 3: Motivators and reasons for cycling). Three-quarters of the cyclists cycled once or less than once a day for recreation (median (IQR) 0.3 (0.1, 0.7)) and for transport (median (IQR) 0.4 (0.2, 1.0)). For recreation, cyclists mostly cycled alone (75%) or with family (61%), and half of them reported cycling for at least 45 min per ride or 14 km per ride. For transport, they usually cycled to shops (80%) or to visit friends (68%), with a median duration of 20 min per ride or five kilometers per ride (Additional file 4: Cycling intensity, frequency, path, and destination according to the purpose of cycling).

### Devices and bicycles used for cycling

Cyclists used their daily prosthesis (33%), daily shoes (82%), and electric bicycle (46%) for cycling. Six participants reported having a specific prosthesis for cycling (4%). Nine percent had an adapted bicycle, and 2% had a prosthesis adapted for cycling. Women’s and men’s bicycles were used by 21% and 12% of cyclists respectively (Additional file 5: Daily prosthesis, walking aids, and shoes of cyclists (n= 141)). There were no differences between the two levels of an amputation (below the knee, and from KD to HD) and types of bicycles (X^2^ = 15.5, *p* = 0.159).

### Facilitators and barriers associated with cycling

Cyclists reported significantly less barriers related to cycling and significantly more facilitators (Table 3). Most frequently mentioned facilitators were having fun/relaxing (recreation) (82%) and increasing or maintaining health/physical fitness (79%) (Additional file 6: Barriers and facilitators).

**Table 3:** Cycling barriers and facilitators reported by cyclists and non-cyclists

	Cyclists (n = 141)		Non-cyclists (n = 66)		Chi-square	*p*
	n	%	n	%		
*Barriers*						
Health: no energy, pain, wound, discomfort, poor health condition	34	24	24	36	3.3	0.067
Negative attitude toward cycling	16	11	18	27	8.3	0.004
Lack of support/encouragement from family, friends, care taker, medical/rehabilitation practitioners, buddies with amputation	0	0	1	2	2.1	0.319
Built environment: dressing room, rest areas, potholes in the road, bike parking space/path	12	9	1	2	3.7	0.066
Safety (crime, speed of cars, number of cars, traffic lights)	5	4	2	3	0.0	1.000
Weather	10	7	2	3	1.4	0.346
Pollution	0	0	0	0		
High expenses for training/bike/prosthesis	3	2	3	5	1.0	0.386
Lack of cycling skills/knowledge of how and where to cycle	2	1	6	9	7.1	0.014
Equipment: no bike/inappropriate bike and/or prosthesis	14	10	21	32	15.3	< 0.001
Distance to destination is too far/too close	2	1	0	0	1.0	0.564
Other	27	19	19	29		
*Facilitators*						
Health: increase/maintain physical fitness, increase/maintain strength, control weight	114	81	21	32	47.7	< 0.001
Attitude: recreation (fun/relaxation), increase/maintain self-confidence, learn new skills, increase/maintain independence, accept disability, learn how to deal with disability/assistive device, increase/maintain social contacts	125	89	19	29	76.1	< 0.001
Support/encouragement from family, friends, caretaker, medical/rehabilitation practitioners, buddies with amputation	18	13	2	3	4.9	0.040
Competition/winning	3	2	0	0	1.4	0.553
Work	22	16	1	2	9.0	0.003
Built environment	37	26	2	3	15.9	< 0.001
Good weather	35	25	2	3	14.5	< 0.001
No pollution	6	4	0	0	2.9	0.180
Safety	31	22	1	2	14.4	< 0.001
Cost and availability of bike/prosthesis	24	17	13	20	0.2	0.698
Knowing how and where to cycle	5	4	3	5	0.1	1.000
Satisfied with a current bike/prosthesis	49	35	3	5	21.8	< 0.001
Appropriate distance to destination	17	12	2	3	4.4	0.039
Other facilitator	8	6	2	3		

Non-cyclists reported more barriers related to health conditions, a negative attitude toward cycling, and a lack of appropriate equipment. They less often reported a positive attitude toward cycling and perceived health benefits (Table 3, Additional file 6: Barriers and facilitators).

### Factors associated with cycling participation in a logistic regression analysis

Logistic regression results showed that the likelihood of cycling with a LLA increased 5.2 times when using a dynamic prosthetic foot compared with a SACH foot. More facilitators increased the cycling likelihood, while the presence of other underlying diseases besides the cause of LLA reduced the cycling likelihood (Table 4). The final model explained 40.2% (Nagelkerke R^2^) of the variance in cycling after a lower limb amputation and correctly classified 82.5% of cases.

**Table 4:** Logistic regression of variables associated with cycling participation

	B	SE	*p*	Exp(B)	95% CI for EXP(B)	
					Lower	Upper
Presence of other underlying disease(s) besides amputation cause	− 0.9	0.429	0.028	0.4	0.2	0.9
Single axis foot^a^	0.4	0.561	0.500	1.5	0.5	4.4
Dynamic foot^a^	1.6	0.481	0.001	5.2	2.0	13.3
Total number of facilitators	0.3	0.069	< 0.001	1.3	1.2	1.5
Constant	− 0.5	0.512	0.351	0.6		

SE: standard error. B: coefficient. Exp (B): odds ratio. CI: confidence interval

^a^Reference category: SACH foot

## Discussion

More than two-thirds of the participants cycled after the LLA. The majority of cyclists already cycled before the LLA. Most of participants were amputated due to a trauma related cause and had a LLA level below the knee joint. None of amputation characteristics; however, were a predictor of cycling.

Cycling participation rate was similar to those reported in a previous study on sport and recreational activities participation in general [4]. Nevertheless, Dutch people with a LLA from this study reported a higher cycling rate than the rate found among people with a LLA in a review (48%) [7] or a survey in Thailand [5]. This difference might be explained by the popularity of bicycle use in the Netherlands [10]. The Netherlands is known as the cycling nation, with more bicycles than inhabitants (23 million bicycles and 17 million inhabitants) [10]. Further people with a LLA cycled for the same reasons as able-bodied people in the Netherlands [10].

Similar to most Thai cyclists who had undergone a LLA, the participants in the current study also initiated cycling themselves and did not rely on healthcare professionals or others for motivation or instruction [5]. Healthcare professionals may not prioritize cycling in the same way as people with a LLA [20]. Many cyclists had a positive attitude toward cycling; they considered it to be fun and good for their health. Another study found that high expectations of the benefits of physical activities were needed to initiate physical activities and that experiencing benefits kept people with a LLA engaged in the activity [21]. Moreover, fun has been reported as an essential factor for maintaining physical activity and was the discriminating factor between older adults who adhere to exercise and those who did not [22].

The majority of cyclists used their walking prosthesis and shoes. Approximately 46% of them used an electric bicycle. Considering that recreation was the main reason for cycling, daily prostheses were likely to provide sufficient function during cycling. Surprisingly, half of the cyclists with a mean age of 59 years cycled longer than 14 km per ride. This is quite a long distance, especially when considering that trips involving distances ranging from 7.5 to 15 km account for only 15% of all bicycle trips [10]. This result can partly be explained by the use of an electric bicycle [23]. Electric bicycles have become increasingly popular in the Netherlands and are mostly used by older adults [10]. The second most popular bicycle among the participants was the women’s bicycle, even though the majority of cyclists in this study were male. Similar to the utility bicycle or grandma bicycle (Dutch: omafiets) that is widely used by Thai cyclists with a LLA [5], “women’s bicycles” have a diagonal frame bar, creating a low instep. Consequently, cyclists do not need to lift their limb over a high horizontal bar, and this can help them get on and off the bicycle with their prosthesis more easily.

### Dynamic foot

A dynamic foot increases the likelihood of cycling, in contrast to a SACH foot [5]. After a LLA, people who have the potential to walk with varying speeds and on various terrains are recommended to use a dynamic foot rather than a SACH foot [24]. This suggests that cyclists may already have a higher functional level in general. In addition, non-cyclists reported more rheumatoid arthritis and diabetes than cyclists. This higher functional level may also explain why non-electric bicycle users were able to cycle further than 40 km per ride.

### Total number of facilitators

Increasing the number of facilitators is important for increasing the likelihood of cycling with a LLA. Cyclists reported a more positive attitude toward cycling and more perceived health benefits than non-cyclists. Although non-cyclists reported fewer facilitators, health benefits and a positive attitude toward cycling were still the most often reported facilitators. These findings suggest that non-cyclists are aware of cycling benefits, but these benefits do not motivate them enough to cycle. Interestingly, although cyclists also reported barriers to cycling such as lack of energy, pain, and discomfort, they continued cycling despite these complaints.

A previous study suggested that lower limb prosthesis users experience more barriers than facilitators when trying to adapt or maintain a physically active lifestyle [25]. In line with that study, non-cyclists in this study also reported more barriers than facilitators, for instance, a lack of skills or knowledge related to cycling or being afraid of getting injured. Most of these barriers hindered them from initiating cycling in the first place. Finding the desire to cycle after a LLA can help tailor the rehabilitation program to the specific needs and physical conditions of the individuals. Consequently, a sense of mastery or accomplishment from the training may facilitate them to continue cycling [25].

Practical factors related to work, built environment, weather, safety, and distance to destination were frequently reported by cyclists. For example, good quality streets, adequate cycling paths, good traffic safety, and good weather. In general, these external factors were frequently reported by cyclists as facilitators and less frequently reported as barriers. This contrasts with the study conducted in Thailand, where these factors were more frequently reported as barriers [2]. These findings suggest that the infrastructure in the Netherlands is quite accommodating to cyclists, with and without a LLA [26].

Presence of other underlying diseases in addition to the cause of LLA.

The presence of underlying diseases predicts that people with a LLA are less likely to cycle. Underlying diseases in addition to a LLA such as rheumatoid arthritis and diabetes can reduce physical activity performance [27, 28]. A study found that, on average, people with diabetes walked less (4603 steps/day) than able-bodied people (7817 steps/day), and people with both diabetes and a transtibial amputation walked even less than people with diabetes (1721 steps/day) [27]. A LLA due to vascular disease negatively associated with sport participation [6]. Another study found that the presence of three or more clinical conditions predicted a worse physical performance in gait speed, chair stand, and balance tests [28]. Consistent with the use of a SACH foot and the reduction in the likelihood of cycling, a negative association between having other underlying diseases and cycling may reflect the lower mobility of the non-cyclist group.

## Limitations

This study has several limitations. First, only 49% of the target sample size participated in the study. The sample size was based on the estimated percentage of participants that were cycling. In that sample we aimed to estimate that percentage with a precision of plus or minus 5%. However, with this sample size it was still possible to estimate cycling participation with a ± 6.5% precision rate. It was not possible to record how many questionnaires were distributed to the potential participants in each facility, which meant that a response rate could not be calculated. A second limitation concerns the fact that the majority of participants in this study had undergone the latest amputation due to trauma. However, vascular diseases are a major cause of LLA in the Netherlands [29]. Many reported other underlying diseases which might be the results of the amputation or lead to the trauma related amputation. This limits the generalizability of this study to other. Third, the accuracy of self-reporting could be influenced by a lack of knowledge of participants, for example, regarding their prosthetic components. This lack of knowledge is illustrated in the lower number of responses regarding prosthetic components used than the number of participants with in that LLA level. Consequently, this missing data may affect the differences among the cyclists and non-cyclists and possibly resulting in a missing predictor in the final model. Information regarding the frequency and duration of cycling, may have been affected by recall bias [30] or the season [31] in which the questionnaire was completed.

## Conclusions

The majority of people continued to cycle after a LLA for recreation, health or transportation purposes. Three important predictors of cycling after a LLA were identified: a dynamic foot, a higher number of facilitators, and no other underlying diseases. Results suggest personal motivation and higher mobility levels have a positive effect on cycling participation. Considering the health benefits of cycling, it is recommended that future studies explore how cycling motivation can be increased in people with LLA and how motivation interacts with mobility levels.

## Supplementary Information

**Additional file 1.** STROBE Statement—Checklist of items that should be included in reports of cross-sectional studies.

**Additional file 2.** Questionnaire (https://doi.org/10.1371/journal.pone.0220649.s001).

**Additional file 3.** Motivators and reasons for cycling.

**Additional file 4.** Cycling intensity, frequency, path, and destination according to the purpose of cycling.

**Additional file 5.** Daily prosthesis, walking aids, and shoes of cyclists (n= 141).

**Additional file 6.** Barriers and facilitators.

## Data Availability

All data were presented in the main manuscript or additional supporting files. The raw data are available upon reasonable request to the corresponding author (JP).

## Abbreviations

STROBE: Strengthening the Reporting of Observational Studies in Epidemiology
LLA: A lower limb amputation
SD: Standard Deviation
IQR: Interquartile range
CI: Confidence interval
kg: Kilogram
cm: Centimeter
m: Meter
HD: Hip disarticulation
TF: Transfemoral
KD: Knee disarticulation
TT: Transtibial
AD: Ankle disarticulation
MF: Midfoot
SACH: Solid ankle cushioned heel
PTB: Patellar tendon bearing
PTBSC: PTB and supracondylar
PTB-SCSP: PTB-SC and suprapatellar
SE: Standard error
B: Coefficient
Exp (B): Odds ratio

## References

[1] Oja P, Titze S, Bauman A, de Geus B, Krenn P, Reger-Nash B (2011). Health benefits of cycling: a systematic review. Scand J Med Sci Sport.

[2] Taddei C, Gnesotto R, Forni S, Bonaccorsi G, Vannucci A, Garofalo G (2015). Cycling promotion and non-communicable disease prevention: health impact assessment and economic evaluation of cycling to work or school in Florence. PLoS ONE.

[3] Götschi T, Garrard J, Giles-Corti B (2016). Cycling as a part of daily life: a review of health perspectives. Transp Rev.

[4] Kars C, Hofman M, Geertzen JHB, Pepping G-J, Dekker R (2009). Participation in sports by lower limb amputees in the Province of Drenthe, The Netherlands. Prosthet Orthot Int.

[5] Poonsiri J, Dekker R, Dijkstra PU, Nutchamlong Y, Dismanopnarong C, Puttipaisan C (2019). Cycling of people with a lower limb amputation in Thailand. PLoS ONE.

[6] Bragaru M, Meulenbelt HEJ, Dijkstra PU, Geertzen JHB, Dekker R (2013). Sports participation of Dutch lower limb amputees. Prosthet Orthot Int.

[7] Poonsiri J, Dekker R, Dijkstra PU, Hijmans JM, Geertzen JHB (2018). Bicycling participation in people with a lower limb amputation: a scoping review. BMC Musculoskelet Disord.

[8] Childers WL, Kistenberg RS, Gregor RJ (2011). Pedaling asymmetries in cyclists with unilateral transtibial amputation: effect of prosthetic foot stiffness. J Appl Biomech.

[9] Childers WL, Kogler GF (2014). Symmetrical kinematics does not imply symmetrical kinetics in people with transtibial amputation using cycling model. J Rehabil Res Dev.

[10] Harms L, Kansen M (2018). Cycling facts. Ministry Infrastruct Water Manag.

[11] Cochran WG (1977). Sampling techniques.

[12] Vandenbroucke JP, Von Elm E, Altman DG, Gøtzsche PC, Mulrow CD, Pocock SJ (2007). Strengthening the reporting of observational studies in epidemiology (STROBE): explanation and elaboration. PLoS Med.

[13] Jaarsma EA, Dijkstra PU, Geertzen JHB, Dekker R (2014). Barriers to and facilitators of sports participation for people with physical disabilities: a systematic review. Scand J Med Sci Sport.

[14] Tsang S, Royse CF, Terkawi AS (2017). Guidelines for developing, translating, and validating a questionnaire in perioperative and pain medicine. Saudi J Anaesth.

[15] Rimmer JH, Wang E, Smith D (2008). Barriers associated with exercise and community access for individuals with stroke. J Rehabil Res Dev.

[16] Jarl GM, Heinemann AW, Norling Hermansson LM (2012). Validity evidence for a modified version of the Orthotics and Prosthetics Users’ Survey. Disabil Rehabil Assist Technol.

[17] Rodrigues IB, Adachi JD, Beattie KA, MacDermid JC (2017). Development and validation of a new tool to measure the facilitators, barriers and preferences to exercise in people with osteoporosis. BMC Musculoskelet Disord.

[18] Brislin RW (1970). Back-translation for cross-cultural research. J Cross Cult Psychol.

[19] Vasudevan V, Rimmer JH, Kviz F (2015). Development of the barriers to physical activity questionnaire for people with mobility impairments. Disabil Health J.

[20] van Schaik L, Hoeksema S, Huvers LF, Geertzen JHB, Dijkstra PU, Dekker R (2020). The most important activities of daily functioning: the opinion of persons with lower limb amputation and healthcare professionals differ considerably. Int J Rehabil Res.

[21] Miller MJ, Jones J, Anderson CB, Christiansen CL (2019). Factors influencing participation in physical activity after dysvascular amputation: a qualitative meta-synthesis. Disabil Rehabil.

[22] Stevens M, Lemmink KA, Heuvelen VM, De Jong J, Rispens P (2003). Groningen Active Living Model (GALM): stimulating physical activity in sedentary older adults; validation of the behavioral change model. Prev Med (Baltim).

[23] Fyhri A, Fearnley N (2015). Effects of e-bikes on bicycle use and mode share. Transp Res D Transp Environ.

[24] Stevens PM, Rheinstein J, Wurdeman SR (2018). Prosthetic foot selection for individuals with lower-limb amputation: a clinical practice guideline. J Prosthetics Orthot.

[25] Deans S, Burns D, McGarry A, Murray K, Mutrie N (2012). Motivations and barriers to prosthesis users participation in physical activity, exercise and sport: a review of the literature. Prosthet Orthot Int.

[26] Hull A, O’Holleran C (2014). Bicycle infrastructure: can good design encourage cycling?. Urban Plan Transp Res.

[27] Paxton RJ, Murray AM, Stevens-Lapsley JE, Sherk KA, Christiansen CL (2016). Physical activity, ambulation, and comorbidities in people with diabetes and lower-limb amputation. J Rehabil Res Dev.

[28] Cesari M, Onder G, Russo A, Zamboni V, Barillaro C, Ferrucci L (2006). Comorbidity and physical function: Results from the aging and longevity study in the Sirente geographic area (iISIRENTE Study). Gerontology.

[29] Fard B, Dijkstra PU, Stewart RE, Geertzen JHB (2018). Incidence rates of dysvascular lower extremity amputation changes in Northern Netherlands: A comparison of three cohorts of 1991–1992, 2003–2004 and 2012–2013. PLoS ONE.

[30] Althubaiti A (2016). Information bias in health research: definition, pitfalls, and adjustment methods. J Multidiscip Healthc.

[31] Heinen E, van Wee B, Maat K (2010). Commuting by bicycle: an overview of the literature. Transp Rev.

