# Supplementary material for: Cycling in people with a lower limb amputation
Source: BMC Sports Sci Med Rehabil. 2021 Jul 10;13:75. doi: 10.1186/s13102-021-00302-3 (PMC8272388; doi:10.1186/s13102-021-00302-3)
Supplement: Supplementary file 2 — Additional file 2. Questionnaire (https://doi.org/10.1371/journal.pone.0220649.s001). [file 13102_2021_302_MOESM2_ESM.docx]

# Additional file 2. Questionnaire (<https://doi.org/10.1371/journal.pone.0220649.s001>)
