# Supplementary material for: Cycling in people with a lower limb amputation
Source: BMC Sports Sci Med Rehabil. 2021 Jul 10;13:75. doi: 10.1186/s13102-021-00302-3 (PMC8272388; doi:10.1186/s13102-021-00302-3)
Supplement: Supplementary file 3 — Additional file 3. Motivators and reasons for cycling. [file 13102_2021_302_MOESM3_ESM.docx]

# Additional file 3. Motivators and reasons for cycling

|  | **N=141** | **%** |
| --- | --- | --- |
| **Motivators:** |  |  |
| I want to ride the bike myself | 124 | 88 |
| Physiotherapist | 34 | 24 |
| Family | 33 | 23 |
| Doctor | 24 | 17 |
| Prosthetist | 13 | 9 |
| Occupational therapist | 9 | 6 |
| Friends | 9 | 6 |
| Other | 8 | 6 |
| Care taker | 1 | 1 |
| Fellow amputees | 1 | 1 |
| **Cycling reasons:** | |  |
| Recreation (Have fun/ relaxation) | 113 | 80 |
| Increase/ maintain health/ physical fitness | 104 | 74 |
| Transport/ commute from one place to another place | 70 | 50 |
| Increase/maintain strength | 65 | 46 |
| Increase independence | 47 | 33 |
| Control weight | 42 | 30 |
| Increase/ maintain social contacts | 31 | 22 |
| Increase self-confidence | 20 | 14 |
| Accept disability | 19 | 14 |
| Learn how to deal with disability/ assistive device | 19 | 14 |
| Work (e.g. deliver some products) | 7 | 5 |
| Learn new skills | 3 | 2 |
| Compete at the national level | 1 | 1 |
| Compete at the international level | 1 | 1 |
| Other reason | 1 | 1 |
