# Supplementary material for: Cycling in people with a lower limb amputation
Source: BMC Sports Sci Med Rehabil. 2021 Jul 10;13:75. doi: 10.1186/s13102-021-00302-3 (PMC8272388; doi:10.1186/s13102-021-00302-3)
Supplement: Supplementary file 4 — Additional file 4. Cycling intensity, frequency, path, and destination according to the purpose of cycling. [file 13102_2021_302_MOESM4_ESM.docx]

# Additional file 4. Cycling intensity, frequency, path, and destination according to the purpose of cycling

|  | | **Cycling during the last 6 months** | | | |
| --- | --- | --- | --- | --- | --- |
|  |  | **Recreation**  **(n= 111/ 141)** | | **Transport**  **(n= 93/ 141)** | |
|  |  | **Median** | **IQR** | **Median** | **IQR** |
| Cycling frequency (times a day) | | 0.3 | (0.1,0.7) | 0.4 | (0.2,1.0) |
| Cycling duration (minutes per ride) | | 45.0 | (30.0,72.5) | 20.0 | (12.3,30.0) |
| Cycling distance (kilometers per ride) | | 14.0 | (9.0,25.0) | 5.0 | (3.0,10.0) |
| Cycling distance of electric bicycle user (kilometers/ ride) | | 13.3 | (10.0,20.0) | 6.0 | (3.0,10.0) |
| Cycling distance of other bicycle user (kilometers/ ride) | | 40.0 | (7.6,58.5) | 5.0 | (2.9,6.4) |
|  | | **n** | **%** | **n** | **%** |
| **Intensity:** | Moderate | 86 | 78 | 81 | 87 |
|  | Vigorous | 15 | 14 | 7 | 8 |
|  | Both intensities | 2 | 2 | 2 | 2 |
| **With:** | Alone | 84 | 76 | NA |  |
|  | Family | 70 | 63 | NA |  |
|  | Friend | 28 | 25 | NA |  |
|  | Amputee | 1 | 1 | NA |  |
|  | Trainer | 1 | 1 | NA |  |
|  | Club member | 8 | 7 | NA |  |
|  | Other | 7 | 6 | NA |  |
| **Where:** | Bike path no car | 85 | 77 | 57 | 61 |
|  | Quiet road | 81 | 73 | 66 | 71 |
|  | Busy road with no bike path | 63 | 57 | 50 | 54 |
|  | Shared path with cars | 57 | 51 | 47 | 50 |
|  | Shared path with pedestrians | 46 | 41 | 29 | 31 |
|  | Footpath | 5 | 5 | 4 | 4 |
|  | Fitness | 18 | 16 | NA |  |
|  | Park | 14 | 13 | NA |  |
|  | Rehabilitation center | 3 | 3 | NA |  |
|  | Other | 13 | 12 | 2 | 2 |
| **Destination:** | Shop |  |  | 76 | 81 |
|  | Visit friends |  |  | 64 | 68 |
|  | School, work |  |  | 18 | 19 |
|  | Train/ bus |  |  | 10 | 11 |
|  | Temple/ church |  |  | 10 | 11 |
|  | Other |  |  | 22 | 24 |

NA= not applicable.
