# Supplementary material for: Cycling in people with a lower limb amputation
Source: BMC Sports Sci Med Rehabil. 2021 Jul 10;13:75. doi: 10.1186/s13102-021-00302-3 (PMC8272388; doi:10.1186/s13102-021-00302-3)
Supplement: Supplementary file 5 — Additional file 5. Daily prosthesis, walking aids, and shoes of cyclists (n= 141). [file 13102_2021_302_MOESM5_ESM.docx]

# Additional file 5. Daily prosthesis, walking aids, and shoes of cyclists (n= 141)

|  |  | **n** | **%** |
| --- | --- | --- | --- |
| **Have cycling prosthesis:** | Yes | 10 | 9 |
|  | No | 106 | 91 |
| **Types of prostheses used:** | Daily prosthesis | 46 | 33 |
|  | Use cycling prosthesis | 6 | 4 |
|  | Adapted prosthesis | 3 | 2 |
|  | Not use prosthesis | 2 | 1 |
|  | Other | 2 | 1 |
| **Type of bicycle used*:** | Electric bicycle | 65 | 46 |
|  | Women’s bicycle | 30 | 21 |
|  | Men’s bicycle | 17 | 12 |
|  | Adapted bicycle | 13 | 9 |
|  | Hand bicycle | 12 | 9 |
|  | Road bicycle | 9 | 6 |
|  | Mountain bicycle | 8 | 6 |
|  | Tricycle | 5 | 4 |
|  | Stationary bicycle | 5 | 4 |
|  | Grandma bicycle | 4 | 3 |
|  | Cargo bicycle | 2 | 1 |
|  | Other | 7 | 5 |
| **Types of shoes used:** | Daily shoes | 116 | 82 |
|  | Daily and cycling shoes | 4 | 3 |
|  | Adapted shoes | 3 | 2 |
|  | Cycling shoes | 2 | 1 |
|  | Other shoes | 6 | 4 |

Data were analyzed using the information reported by people who cycled. *39 cyclists had more than 1 type of bicycle.
