# Supplementary material for: Cycling in people with a lower limb amputation
Source: BMC Sports Sci Med Rehabil. 2021 Jul 10;13:75. doi: 10.1186/s13102-021-00302-3 (PMC8272388; doi:10.1186/s13102-021-00302-3)
Supplement: Supplementary file 6 — Additional file 6. Barriers and facilitators. [file 13102_2021_302_MOESM6_ESM.docx]

# Additional file 6: Barriers and facilitators

|  | Cyclist  (n=141 ) | | Non-cyclists  (n=66) | |
| --- | --- | --- | --- | --- |
|  | **n** | **%** | **n** | **%** |
| Barriers: |  |  |  |  |
| Lack of energy/effort | 12 | 8.5 | 13 | 19.7 |
| Pain | 15 | 10.6 | 6 | 9.1 |
| Wound/Injury | 6 | 4.3 | 1 | 1.5 |
| Discomfort while cycling | 12 | 8.5 | 9 | 13.6 |
| Poor health conditions | 1 | 0.7 | 5 | 7.6 |
| Lack of time | 5 | 3.5 | 0 | 0 |
| Lack of motivation | 2 | 1.4 | 1 | 1.5 |
| Afraid of being injured | 6 | 4.3 | 15 | 22.7 |
| Feeling embarrassed about my appearance while cycling | 1 | 0.7 | 0 | 0 |
| Lack of health improvement from cycling | 2 | 1.4 | 1 | 1.5 |
| Feeling too old to cycle | 1 | 0.7 | 4 | 6.1 |
| Lack of fun from cycling | 2 | 1.4 | 3 | 4.5 |
| Lack of reasons to cycle | 1 | 0.7 | 3 | 4.5 |
| Lack of family members who are cycling | 0 | 0 | 1 | 1.5 |
| Lack of friends who are cycling | 0 | 0 | 1 | 1.5 |
| Lack of support/encouragement from friends/family/ care taker | 0 | 0 | 0 | 0 |
| Lack of support/encouragement from medical/rehabilitation practitioners | 0 | 0 | 0 | 0 |
| Lack of access to dressing rooms (changing clothes/having a shower) | 0 | 0 | 0 | 0 |
| Lack of rest areas (e.g., benches) | 0 | 0 | 0 | 0 |
| Potholes in the street | 11 | 7.8 | 1 | 1.5 |
| Lack of parking for bicycle | 1 | 0.7 | 0 | 0 |
| Lack of cycling paths/lanes | 2 | 1.4 | 1 | 1.5 |
| Excessive crime in neighborhood or fear of crime in neighborhood | 0 | 0 | 0 | 0 |
| Cars driving too fast on the road | 4 | 2.8 | 1 | 1.5 |
| Excessive car traffic in my community | 0 | 0 | 1 | 1.5 |
| Lack of traffic lights or cross signals for cycling | 0 | 0 | 0 | 0 |
| Lack of adequate street lighting at night | 0 | 0 | 0 | 0 |
| Loose dogs in community | 1 | 0.7 | 0 | 0 |
| Bad weather (hot, rain) | 10 | 7.1 | 2 | 3.0 |
| Pollution | 0 | 0 | 0 | 0 |
| High costs of cycling equipment | 2 | 1.4 | 3 | 4.5 |
| High costs of cycling prosthesis or high costs to adapt prosthesis | 2 | 1.4 | 2 | 3.0 |
| High costs of cycling training | 0 | 0 | 1 | 1.5 |
| Lack of knowledge or skills on how to cycle before the amputation | 1 | 0.7 | 2 | 3.0 |
| Lack of knowledge or skills on how to cycle after the amputation | 1 | 0.7 | 5 | 7.6 |
| Lack of information on where to cycle | 0 | 0 | 0 | 0 |
| Not owning a bicycle | 3 | 2.1 | 9 | 13.6 |
| Daily prosthesis problems; prosthesis prevents me from cycling | 9 | 6.4 | 14 | 21.2 |
| Bicycle problems; the bicycle is not suitable for conditions | 2 | 1.4 | 2 | 3.0 |
| Too close to cycle to destination | 0 | 0 | 0 | 0 |
| Too far to cycle to destination | 2 | 1.4 | 0 | 0 |
| Other barriers | 27 | 19.1 | 19 | 28.8 |
| Facilitators: |  |  |  |  |
| Increasing / maintaining health/physical fitness | 112 | 79.4 | 19 | 28.8 |
| Increasing/ maintaining strength | 82 | 58.2 | 14 | 21.2 |
| Controlling weight | 48 | 34.0 | 10 | 15.2 |
| Having fun/ relaxation | 116 | 82.3 | 17 | 25.8 |
| Increasing/maintaining self-confidence | 24 | 17.0 | 3 | 4.5 |
| Learning new skills | 9 | 6.4 | 3 | 4.5 |
| Increasing/maintaining independence | 38 | 27.0 | 7 | 10.6 |
| Accepting disability | 18 | 12.8 | 5 | 7.6 |
| Learning how to deal with disability/ assistive device | 15 | 10.6 | 3 | 4.5 |
| Increasing/ maintaining social contacts | 38 | 27.0 | 5 | 7.6 |
| Support/encouragement from family | 13 | 9.2 | 0 | 0 |
| Support/encouragement from friends | 5 | 3.5 | 1 | 1.5 |
| Support/encouragement from personal care taker | 1 | 0.7 | 0 | 0 |
| Support/encouragement from medical/rehabilitation practitioners | 6 | 4.3 | 2 | 3.0 |
| Support/encouragement from buddies with amputation | 3 | 2.1 | 0 | 0 |
| Competition/winning | 3 | 2.1 | 0 | 0 |
| Work | 22 | 15.6 | 1 | 1.5 |
| Adequate dressing rooms (changing clothes/ shower) | 1 | 0.7 | 0 | 0 |
| Adequate rest areas (e.g., benches) | 8 | 5.7 | 1 | 1.5 |
| Good quality streets - no potholes | 22 | 15.6 | 1 | 1.5 |
| Adequate parking for bicycles | 10 | 7.1 | 0 | 0 |
| Adequate cycling paths/ lanes | 24 | 17.0 | 0 | 0 |
| Safe neighborhoods - low crime | 14 | 9.9 | 0 | 0 |
| Cars driving with appropriate speed on the road/not too fast | 4 | 2.8 | 0 | 0 |
| Good traffic/not many cars on the road | 15 | 10.6 | 1 | 1.5 |
| Adequate traffic lights or cross signals for bicycle | 8 | 5.7 | 0 | 0 |
| Adequate street lighting at night | 7 | 5.0 | 0 | 0 |
| Good weather | 35 | 24.8 | 2 | 3.0 |
| No pollution | 6 | 4.3 | 0 | 0 |
| Affordable costs of cycling equipment/accessories | 10 | 7.1 | 5 | 7.6 |
| Affordable costs of cycling prosthesis/adapted prosthesis | 10 | 7.1 | 6 | 9.1 |
| Affordable costs of cycling/ training program | 2 | 1.4 | 2 | 3.0 |
| Free adapted/prosthesis for cycling | 8 | 5.7 | 6 | 9.1 |
| Free adaptation of bicycle | 10 | 7.1 | 5 | 7.6 |
| Free cycling training | 2 | 1.4 | 6 | 9.1 |
| Knowing how to cycle | 2 | 1.4 | 3 | 4.5 |
| Knowing where to cycle | 4 | 2.8 | 1 | 1.5 |
| Good satisfaction with daily prosthesis | 34 | 24.1 | 1 | 1.5 |
| Having a bicycle that fits with my conditions | 30 | 21.3 | 3 | 4.5 |
| Appropriate distance to destination/not too far or too close | 17 | 12.1 | 2 | 3.0 |
| Other facilitators | 8 | 5.7 | 2 | 3.0 |
